# Artificial Intelligence in Chronic Rhinosinusitis: From Molecular Profiling to Personalized Clinical Management—A Systematic Review

**DOI:** 10.3390/ijms27198812

**Published:** 2026-10-01

**Authors:** Chi-Sheng Huang, Duen-Lii Hsieh, Jin-Yu Lin, Maysayawan Sreesawat, Te-Huei Yeh, Chih-Feng Lin

**Affiliations:** 1 School of Medicine, National Taiwan University, Taipei 100233, Taiwan; b09401036@ntu.edu.tw; 2 Department of Otolaryngology Head and Neck Surgery, National Taiwan University Hospital, Taipei 100225, Taiwan; dtent86@yahoo.com.tw; 3 Department of Computer Science and Information Engineering, National Cheng Kung University, Tainan 701301, Taiwan; f74141068@gs.ncku.edu.tw; 4 Department of Otorhinolaryngology, Faculty of Medicine Siriraj Hospital, Mahidol University, Bangkok 10700, Thailand; maysayawan@gmail.com; 5 Department of Otolaryngology Head and Neck Surgery, National Taiwan University Hospital Hsin-Chu Branch, Hsinchu 300195, Taiwan; 6 Institute of Medical Device and Imaging, National Taiwan University, Taipei 100233, Taiwan

**Keywords:** chronic rhinosinusitis, artificial intelligence, machine learning, deep learning, precision medicine, molecular endotypes

## Abstract

Chronic rhinosinusitis (CRS) is a heterogeneous inflammatory disease imposing a substantial health and economic burden. Artificial intelligence (AI) has emerged as a promising tool to address CRS complexity in both clinical and research settings. We conducted a systematic review following PRISMA guidelines, searching PubMed, Web of Science, and Embase for peer-reviewed articles utilizing machine learning, deep learning, and natural language processing, with a focus on molecular-level or immunologic mechanisms in CRS. Thirty-one studies were included in the qualitative synthesis. Our results demonstrate that while AI-driven analysis helps stratify patient endotypes, the identified schemes exhibit substantial heterogeneity in cluster numbers and features, raising concerns about their reproducibility. AI applications also included histopathological image interpretation, drug discovery, biologic therapy selection, and prognostic modeling to evaluate revision surgery risks and olfactory dysfunction. However, using the PROBAST+AI framework, we found that 87.1% of the assessed studies carry a high overall risk of bias, mainly due to the reliance on apparent performance or internal validation and limited independent evaluation of model performance, indicating that many reported performance estimates may be optimistic. Future large-scale, multicenter collaborations integrating AI into clinical workflows will be essential to establish reliable and generalizable AI applications for personalized CRS management.

## 1. Introduction

**Figure 1:**
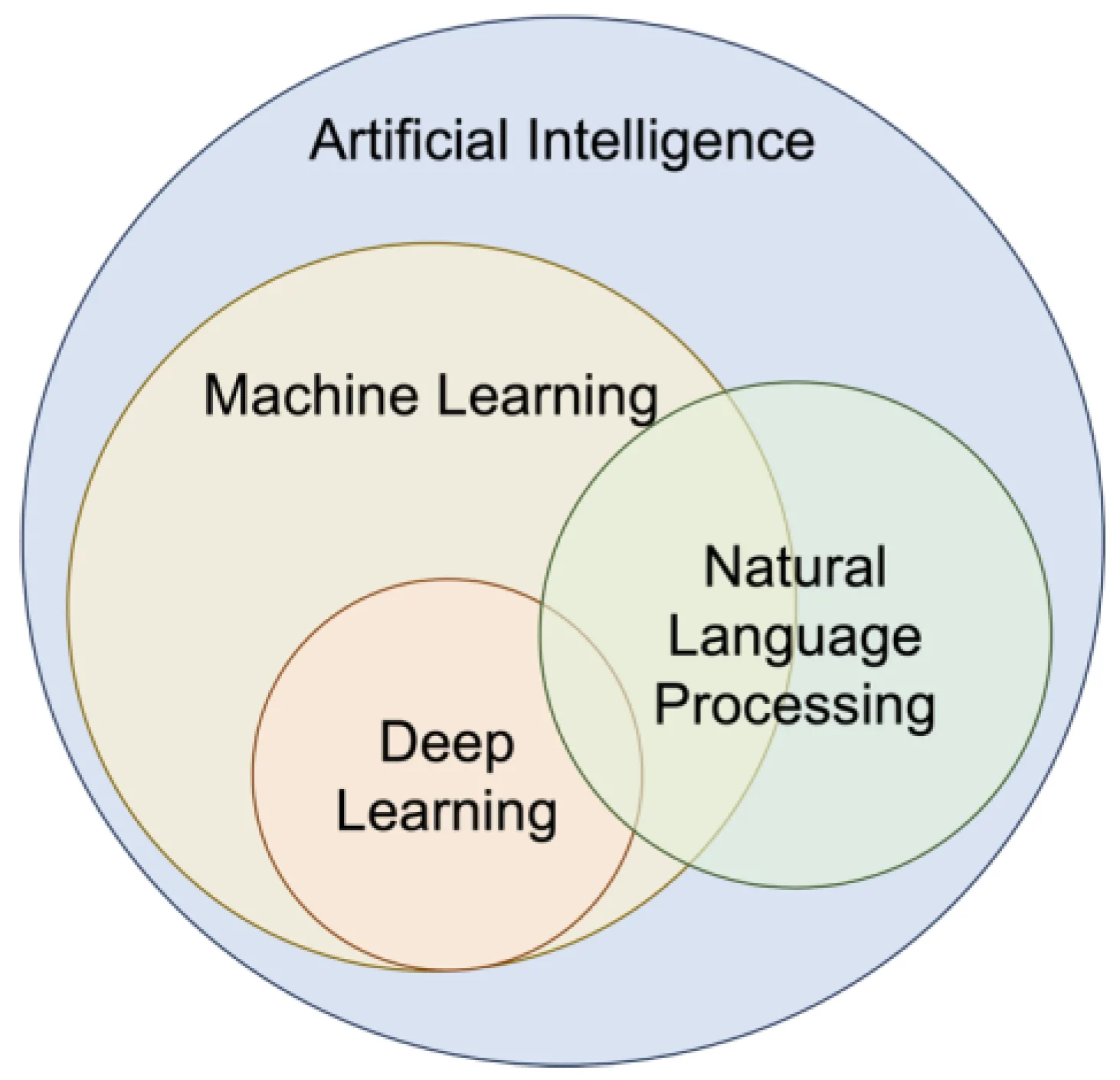
Relationship between machine learning (ML), deep learning (DL), and natural language processing (NLP). ML is algorithms in AI that learn patterns from data. DL is a subfield of ML based on artificial neural networks (ANNs). NLP applies ML and DL techniques to analyze and represent human language.

Artificial Intelligence (AI) brings about many benefits as it allows machines to perform tasks that traditionally require human intelligence, including reasoning, pattern recognition, decision-making, and language comprehension. Recent breakthroughs in machine learning (ML), deep learning (DL), and natural language processing (NLP) have enabled AI systems to process complex datasets, perform tasks automatically, and generate personalized responses, which will open the door to revolutionary applications across a range of medical areas, such as disease diagnosis, clinical treatment, and medical research, among others [1,2]. In the early 1970s, MYCIN was designed to assist physicians in diagnosing and treating bacterial infections, marking a milestone in the AI application of medicine [3]. After that, the rapid advancement of computer science has led to the development of various AI technologies. Research has shown potential applications of AI in cancer screening and research, detecting diabetic retinopathy, EKG reading, and chest X-ray interpretation [4,5,6,7]. Regarding disease management, AI holds promise in the precision medicine of chronic disease, the prediction of individual response to cancer treatment, and rational drug dosage and monitoring in clinical practice [8,9,10,11]. The relationship between these AI techniques is demonstrated in Figure 1.

Table 1 shows a comparison of ML, DL, and NLP. ML provides the foundational approaches, DL advances automated feature extraction for complex data, and NLP applies these methods to human language understanding. A rapidly evolving application of NLP with DL is the development of large language models, among which ChatGPT is one of the most prominent examples [12].

**Table 1:** Comparison of machine learning (ML), deep learning (DL), and natural language processing (NLP) [12,13,14,15,16].

Algorithm	ML	DL	NLP
Definition	Algorithms that learn patterns from data, including supervised, unsupervised, semi-supervised, and reinforcement learning	A subfield of ML based on artificial neural networks (ANNs), using multilayer nodes to automatically extract features	Applies ML and DL techniques to analyze and represent human language, enabling models to extract meaning from text or speech
Data characteristics	Labeled, unlabeled, or mixed datasets	Especially suitable for big data in terms of volume, velocity, and variety	Data mainly comes from language, including text or speech
Learning methods	-Supervised: learns a mapping function -Unsupervised: finds hidden structures -Semi-supervised: analyzes a combination of labeled and unlabeled data -Reinforcement: learns through interaction and rewards	Uses multilayer artificial neural networks (ANNs) for automatic feature learning	Uses word co-occurrence for syntax, symbolic processing for semantics, and cognitive emulation to resolve ambiguity
Clinical applications	Disease classification [17,18], diagnosis [19], risk stratification [20], treatment regimen planning [21], disease trajectories prediction [22]	Medical image interpretation [7], early disease diagnosis [23]	Electronic medical record analysis [24], clinical trial recruitment [25]

Chronic rhinosinusitis (CRS) is a significant health issue, impacting approximately 5–12% of the general population [26]. The risk factors of CRS include genetic predisposition, environmental factors, and comorbid conditions such as asthma and allergies [27]. The nasal and facial symptoms are more prominent than systemic or oropharyngeal symptoms [28]. The chronicity leads to substantial medical resource utilization and a global economic burden, driven mainly by increased office visits and prescription costs, with national healthcare expenditures in the U.S. being estimated at $8.6 billion annually [29], and €1501 per patient based on a Dutch cohort [30]. CRS is a heterogeneous disease known by its diverse underlying pathophysiology and classification. By phenotypic classification, CRS can be categorized based on endoscopic features, clinical courses, and comorbidities. For example, CRS has been traditionally divided into CRS with nasal polyps (CRSwNP) and CRS without nasal polyps (CRSsNP) according to the presence of nasal polyps [26]. Recent research has shifted focus to endotyping, which categorizes CRS based on the underlying pathophysiological mechanisms [31]. The T helper cell responses and their downstream effects in CRS have led to the identification of three distinct endotypes, namely the Type 1, Type 2, and Type 3 endotypes, which can be broadly categorized into Type 2 and non-Type 2 endotypes based on phenotype prevalence, clinical comorbidities, therapeutic targets, and response to treatment, where various biologics such as dupilumab, omalizumab, and mepolizumab have been developed in recent years [32,33,34]. The European Position Paper on Rhinosinusitis and Nasal Polyps (EPOS) 2020 introduced a revised classification system for CRS, with a combination of clinical features, localized or diffuse presentations, endotype, and phenotype [26,35]. Table 2 summarizes the common classification frameworks of CRS.

Clinical management of CRS has faced multiple challenges due to its marked disease heterogeneity, complicated classification frameworks, diverse treatment modalities, and varied patient profiles [9,36]. These make CRS a strong candidate for AI integration, as AI offers a powerful solution by integrating and analyzing large-scale clinical and molecular datasets. This review summarizes the current application and the future perspectives of AI in the field of CRS.

## 2. Methods

We performed a structured systematic review according to PRISMA guidelines [37] (the completed PRISMA checklist is available in Supplementary Table S1). The protocol for this review was not registered. A systematic literature search in PubMed, Web of Science, and Embase databases from 1 January 2015 to 30 May 2025 was conducted to identify peer-reviewed articles that had employed AI approaches in CRS diagnosis, classification, imaging, pharmacology, surgery, and prognostication. The search strategy applied keywords and MeSH terms related to “chronic rhinosinusitis,” “rhinosinusitis,” and “chronic sinusitis.” AI-related terms included “artificial intelligence,” “AI,” “machine learning,” “deep learning,” “data mining,” “neural network,” “supervised,” “unsupervised,” “cluster,” “natural language processing,” “ChatGPT,” “large language model,” and “predictive model.” The full search strategies for all databases are provided in Supplementary Table S2. Boolean operators (“AND,” “OR”) were used to combine disease-related and AI-related terms. The final literature search was conducted on 10 June 2025.

All identified records were imported into EndNote 2025 (Clarivate Analytics, Philadelphia, PA, USA) for reference management. We utilized both an automation tool on EndNote and manual checking to remove duplicate records. Two independent reviewers screened the titles and abstracts during the initial screening phase. We excluded records that did not focus on CRS, did not utilize AI-based approaches, lacked a molecular-level or immunologic mechanistic focus, were non-English language publications, or were review articles or commentaries. Full-text articles of the remaining records were assessed for eligibility by the same reviewers. Reports were further excluded from the final synthesis if they demonstrated a lack of AI model performance or validation metrics, presented duplicate data or cohort overlap, or contained insufficient AI methodological details. Any discrepancies between the two reviewers during the screening and selection process were resolved through discussion and consensus, or by consulting a third senior reviewer.

Data extraction was performed independently by two reviewers. The extracted data items included: first author, publication year, specific clinical task, data modality and size, type of AI or machine learning algorithms, validation methods, evaluation metrics, and main outcomes. The primary outcomes sought were the predictive performance of the AI models and the identified clinical or biological features. When multiple models were evaluated within a single study, the results of the best-performing model were prioritized for extraction. Regarding missing or unclear information, no assumptions were made, and only available data were extracted and synthesized.

The methodological quality and risk of bias of the included studies were appraised using the newly updated Prediction model Risk Of Bias assessment Tool for AI (PROBAST+AI) framework [38]. In accordance with the updated framework, two reviewers independently assessed each study across two distinct phases: the methodological quality of the model development process, and the risk of bias in the evaluation of model performance. Both phases were assessed across four signaling domains: participants and data sources, predictors, outcomes, and analysis. Applicability was assessed separately for participants and data sources, predictors, and outcomes. Disagreements in the quality assessment were resolved by consensus. PROBAST+AI distinguishes apparent, internal, and external validation, noting that the absence of external performance evaluation may lead to a high Domain 4 rating. However, in accordance with the PROBAST+AI framework, an overall high risk of bias may be reclassified to low when the model was developed on a large dataset, appropriately internally validated, and the other domains showed low concerns.

In accordance with PRISMA 2020 guidelines (Item 4) and the PROBAST+AI framework, our review objective and applicability assessments were anchored to the following PICOTS criteria: Population (P): Adult patients diagnosed with chronic rhinosinusitis (CRSwNP or CRSsNP). Index model (I): Artificial intelligence or machine learning algorithms investigating molecular-level or immunologic mechanisms through the analysis of molecular, immunological, digital pathology, or related clinical surrogate data. Comparator (C): Traditional statistical predictive models or no comparative model. Outcome (O): Identification of disease endotypes, histopathological classifications, and prediction of clinical outcomes. Timing & Setting (T, S): preoperative, intraoperative, or postoperative diagnostic and prognostic settings in tertiary or general clinical environments. Given the nascent and rapidly evolving nature of AI applications in rhinology, our PICOTS criteria were intentionally designed to be comprehensive rather than narrowly restricted. This approach enables us to capture the full landscape of how machine learning is currently being deployed across different biological modalities (e.g., transcriptomics, proteomics, and digital pathology) to address the clinical heterogeneity of CRS. Consequently, due to the significant heterogeneity in the clinical tasks, data modalities, and performance metrics across the included studies, a quantitative meta-analysis was not feasible. Instead, a narrative synthesis was performed in adherence to the Synthesis Without Meta-analysis (SWiM) reporting guidelines to ensure a transparent evaluation [39]. The characteristics and main outcomes of the individual studies were tabulated to facilitate visual comparison, and the extracted data were systematically categorized and presented according to their specific clinical applications in CRS.

## 3. Results

### 3.1. Study Selection

**Figure 2:**
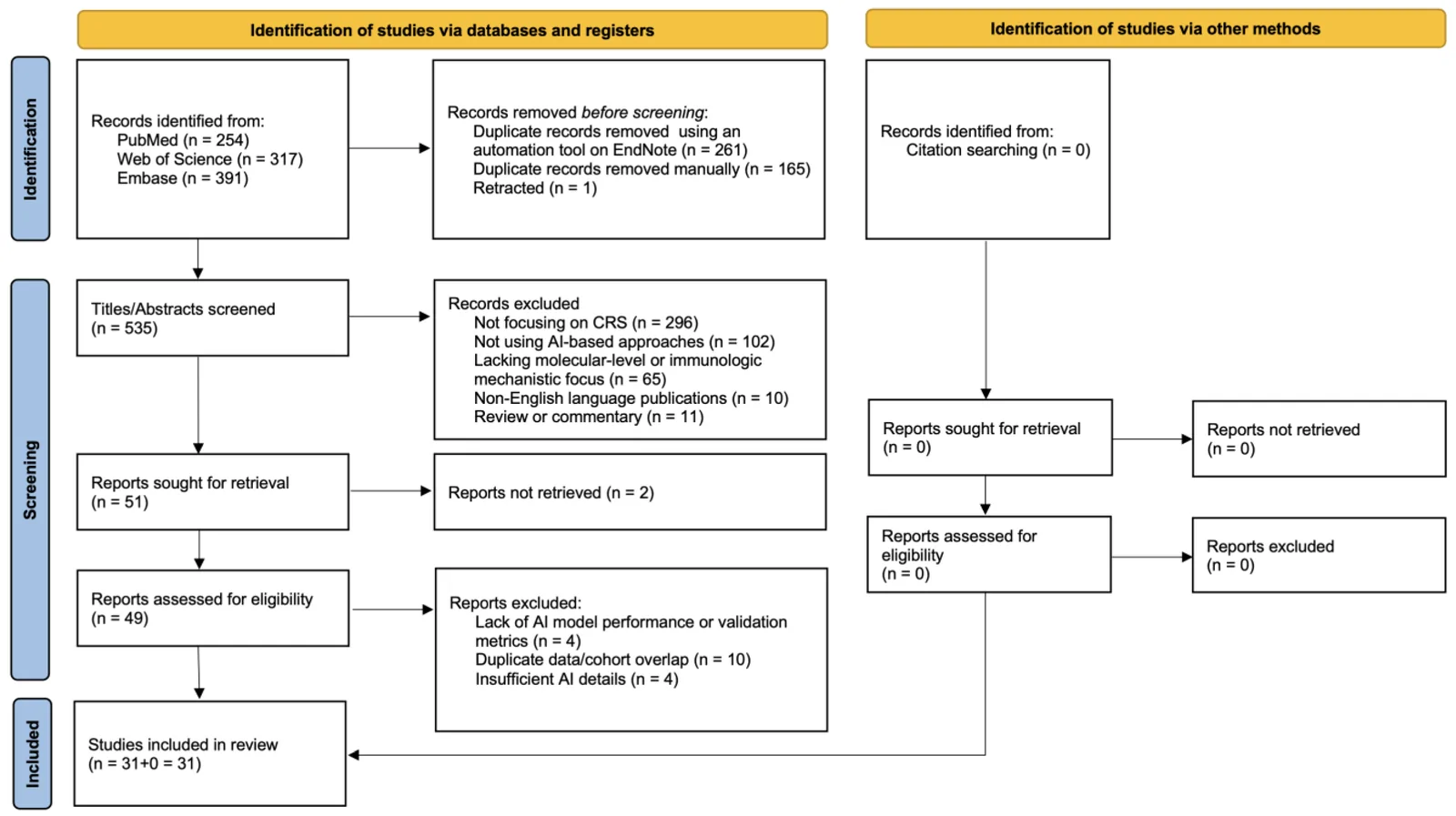
PRISMA 2020 flow diagram illustrating the selection process of studies for the systematic review, including records identification, screening, and exclusion criteria.

Figure 2 illustrates the detailed selection process. A total of 962 records were identified, including 254 from PubMed, 317 from Web of Science, and 391 from Embase, and imported into EndNote for reference management. 427 records were removed, consisting of 261 duplicates identified using an automation tool in EndNote, 165 duplicates removed manually, and 1 retracted record. 535 records were screened by title and abstract. Among them, 484 were excluded for not meeting the following inclusion criteria: not focusing on CRS (n = 296), not using AI-based approaches (n = 102), lacking a molecular-level or immunologic mechanistic focus (n = 65), being review articles or commentaries (n = 11), and being non-English language publications (n = 10). The remaining 51 reports were sought for retrieval and underwent full-text assessment for eligibility, while 2 reports were not retrieved due to unavailable full texts or access restrictions. The remaining 49 full-text articles were assessed for eligibility, and 18 reports were further excluded due to duplicate data or cohort overlap (n = 10), a lack of AI model performance or validation metrics (n = 4), and insufficient AI methodological details (*n* = 4). No additional records were identified through citation searching or other methods. Finally, 31 studies were included in the qualitative synthesis.

### 3.2. Study Characteristics

Table 3 summarizes the characteristics and AI applications of included studies. The 31 included studies consisted of 11 (35.5%) primary prospective studies, 9 (29.0%) cross-sectional studies, 5 (16.1%) retrospective analyses, 4 (12.9%) in silico database analyses, and 2 (6.5%) observational/case–control studies. Because several studies incorporated more than one data modality, the following categories were not mutually exclusive. Thirteen studies (41.9%) incorporated clinical features, including structured clinical records, olfactory test results, and symptom scoring systems such as the SNOT-22. Twelve studies (38.7%) analyzed non-invasive biomarkers, utilizing inflammatory mediators derived from nasal mucus, secretions, serum, urine, and nasal mucus-derived exosomes (NMDEs). Six studies (19.4%) used transcriptomic and multi-omics data, containing single-cell RNA sequencing, bulk RNA sequencing, and microarray profiling from public databases. Three studies (9.7%) applied automated digital pathology, utilizing whole slide images (WSIs) and H&E-stained sections for immune cell quantification.

**Table 3:** Summary of characteristics and artificial intelligence applications of included studies.

Author, Year	Clinical Task	Study Design & Data Modality	Type of Algorithm	Validation Method	Evaluation Metrics	Main Outcomes
Adnane et al., 2017 [40]	Identify endotypes to predict QOL outcomes after surgery.	Prospective cohort study; clinical factors (nasal polyps, gender, mucosal eosinophilia) and SNOT-22 scores from 113 patients.	Unsupervised two-step clustering method.	Internal validation.	*p*-values.	Identified 3 clusters; the cluster with the highest mucosal eosinophilia endotype had the worst QOL improvement after surgery.
Becker et al., 2025 [41]	Predict CRS phenotypes (CRSwNP vs. CRSsNP) using non-invasive mucus biomarkers.	Prospective study; 12 cytokines/inflammatory protein biomarkers from the nasal mucus of 103 CRS patients.	t-SNE, Logistic regression, Decision tree, Support Vector Machine, Adaboost, and XGBoost.	Internal validation (10 iterations of an 80:20 train–test data split).	Accuracy, Adjusted *p*-values.	IL-5 was the main trigger for clustering; the combination of IL-5, IgE, IL-17, and periostin showed the highest prediction accuracy.
Chen J. et al., 2022 [42]	Identify ceRNA regulatory networks and immune infiltration patterns.	In silico transcriptomic analysis; lncRNA, miRNA, and mRNA expression profiles from the Gene Expression Omnibus (GEO).	Bioinformatics data mining and CIBERSORT analysis (SVR-based algorithm).	In-silico dataset validation and experimental validation (immunohistochemistry, immunofluorescence, and Western blot).	AUC.	Identified the MIAT/miR-125a/IRF4 axis; MIAT and IRF4 achieved an AUC of 0.944 and were correlated with specific immune cells.
Chen Z. et al., 2025 [43]	Identify anoikis-related molecular subtypes and key biomarkers.	In silico transcriptomic analysis; 338 anoikis-related genes from the GSE136825 and GSE179625 GEO datasets.	Consensus clustering, LASSO, SVM-RFE, and Random Forest.	Internal validation and Experimental validation (qRT-PCR, IHC, IF).	AUC, *p*-values.	Stratified patients into 2 anoikis-related clusters; identified 4 key genes forming a nomogram model with an AUC >0.90.
Chowdhury et al., 2020 [44]	Predict postoperative SNOT-22 score improvement using baseline biomarkers.	Prospective longitudinal cohort study; 17 intraoperative mucus inflammatory mediators and SNOT-22 scores from 147 CRS patients, 62 with longitudinal postoperative follow-up	Random Forest algorithm and Stepwise multivariate linear regression.	Internal validation.	Beta coefficient, *p*-values, Adjusted R-squared.	The random forest model identified IL-5, TNF-alpha, IL-13, and IL-2 as major predictors of postoperative SNOT-22 scores.
Divekar et al., 2015 [45]	Characterizing local and systemic immune signatures during acute exacerbations of CRSwNP.	Prospective non-interventional study; 39 serum cytokines/ biomarkers, nasal secretions, and SNOT-22 scores from 9 CRSwNP patients and 10 healthy controls during acute exacerbation and baseline.	Unsupervised network analysis and Agglomerative hierarchical clustering.	Internal validation.	*p*-values, Weighted-degree centrality, Aggregate edge weight.	CRSwNP patients exhibit distinct systemic increases in VEGF and GM-CSF during acute exacerbations, correlating with unique network signatures.
Divekar et al., 2017 [46]	Endotype chronic rhinosinusitis using simplified cytokine assays.	Cross-sectional study; 41-plex cytokine–chemokine array and MBP assay on sinonasal surgical tissue from 32 adults (26 CRS, 6 controls).	Unsupervised network modeling and Bipartite network graph clustering.	Mathematical validation of network distances via association index.	Association index, *p*-values.	Revealed 3 analyte clusters and 3 subject groups that distinctively endotyped CRS patients and controls.
Dorismond et al., 2025 [47]	Compare disease trajectory (time-to-polyp recurrence) between CRSwNP endotypes.	Prospective cohort study; 17 baseline mucus cytokines from 269 CRSwNP patients.	PCA and Hierarchical cluster analysis.	Internal validation.	Eigenvalues, Hazard Ratios, and *p*-values.	Identified 6 clusters; mixed type 1/3 and IL-12/21 clusters showed the shortest time to recurrence, whereas the innate proinflammatory cluster had the longest.
Guo et al., 2021 [48]	Predicting “difficult-to-treat” chronic rhinosinusitis before endoscopic sinus surgery	Prospective cohort study; 39 biological markers from noninvasive nasal secretions (n = 189) and peripheral blood (n = 128), combined with baseline clinical features to predict 1-year postoperative CRS outcomes.	Classification and Regression Tree and Random Forest.	Internal validation.	Accuracy, Mean Decrease Gini index, Odds ratio, *p*-values.	A combination of high nasal secretion CCL17 and MIP-1β levels, severe hyposmia, and allergic rhinitis accurately predicts difficult-to-treat CRS with 94% accuracy.
Hsu et al., 2024 [49]	Automated eosinophil quantification in sinonasal histopathology images.	Retrospective image dataset analysis; whole slide images (WSIs)/sinonasal histopathology images.	Multiple instance learning and Hierarchical framework.	Internal validation.	Accuracy.	Successfully estimated eosinophil quantities with a high degree of accuracy and handled cellular distribution variability in WSIs.
Ishino et al., 2024 [50]	Evaluate PAF-metabolism-associated gene expression in CRSwNP subtypes.	Cross-sectional transcriptomic analysis; transcriptomic data using bulk RNA barcoding and sequencing from 26 subjects.	PCA and Hierarchical cluster analysis.	Internal validation	Fold change, False Discovery Rate, *p*-value.	Identified 2 clusters reflecting low-to-moderate and severe type 2 inflammation with distinct regulation of PAF-metabolism-associated genes.
Kim et al., 2021 [51]	Predict surgical outcomes of CRSwNP focusing on subepithelial neutrophil infiltration.	Retrospective cohort study; immunofluorescence data (HNE, Bcl-2, Ki-67) from nasal polyp tissues and clinical scores (Lund–Mackay scores, age).	Binary decision tree and Random Forest.	Internal validation.	*p*-values, Mean squared error, Variable importance scores.	Decision tree identified subepithelial HNE-positive cells, LM scores, and endotypes as top predictors; random forest ranked LM score, age, and subepithelial HNE-positive cells as top predictors for surgical outcomes.
Li et al., 2024 [52]	Predict surgical outcomes for non-eosinophilic CRSwNP patients.	Prospective cohort study; clinical information (SNOT-22) and a broad spectrum of biomarkers in tissue homogenates from 51 patients.	Random Forest and Stepwise logistic regression.	Internal validation using the bootstrapping method.	Gini coefficient, AIC, AUC, ROC curve, Youden index.	Identified SNOT-22 and 4 biomarkers (PDGF-β, programmed cell death ligand 1, macrophage inflammatory protein-3b, PDGF-α) as predictors; combined model achieved an AUC of 0.89.
Liao et al., 2018 [53]	Identify multidimensional CRS endotypes and explore their association with treatment outcomes.	Prospective cohort study; 28 clinical variables and 39 mucosal cellular/molecular variables from 246 prospectively recruited patients and 16 control subjects.	PCA and Unsupervised k-means cluster analysis.	Internal validation.	*p*-values, Eigenvalues.	Identified 7 clusters (e.g., severe eosinophilic, neutrophilic, low inflammation); difficult-to-treat CRS was associated with distinct clinical features and biomarkers across different clusters.
Liu et al., 2022 [54]	Quantify microvessels to explore the association between angiogenesis and CRS inflammation.	Prospective cross-sectional study; 552 hematoxylin and eosin (H&E)-stained images of 27 CRS tissue samples, and tissue homogenates from 79 CRS patients and 17 controls.	Fully convolutional network.	Internal validation (Slide-level Train/Validation/Test split).	Pixel accuracy, mIoU, R^2^ values, *p*-values.	Angiogenesis was higher in type 2 CRS; microvessel area ratio negatively correlated with TNF-alpha and TGF-beta, and positively correlated with IL-5 and ECP.
Lou et al., 2016 [55]	Identify cellular phenotypes of CRSwNP and define a predictive algorithm for polyp recurrence.	Retrospective cohort study; 18 variables: clinical characteristics, tissue/peripheral inflammatory cells from 366 patients.	Factor analysis, Unsupervised cluster analysis, and Discriminant analysis.	Internal validation and Prognostic validation.	Prediction accuracy, Recurrence rate.	Identified 5 phenotypic clusters; cluster 5 (marked tissue eosinophilia) had the highest recurrence rate (98.5%). The discriminant algorithm predicted clustering with 93.7% accuracy.
Miyake et al., 2019 [56]	Predict CRS phenotype and disease severity using noninvasive exosomal cystatins.	Cross-sectional cohort study; nasal mucus-derived exosomes (NMDEs) and clinical variables from 105 patients.	Linear discriminant analysis and Unsupervised cluster analysis.	Internal validation.	Accuracy, Canonical correlation coefficients, *p*-values.	Identified 7 clusters; highest NMDE Cystatin-2 levels clustered with asthma, tissue eosinophilia, and AERD.
Morse et al., 2019 [57]	Validate patterns of olfactory dysfunction and identify predictive cytokines.	Cross-sectional cohort study; Smell Identification Test scores and 17 mucus inflammatory mediators from 110 patients.	Hierarchical cluster analysis and Random Forest.	Internal validation (Elbow method and Bootstrap analysis for clustering and 50/50 train-validation split for random forest).	Sum of squared error, Percent variance explained, *p*-values.	Identified 5 distinct clusters; anosmic patients were mostly in a cluster with severe polyposis and AERD. Random forest identified IL-5 and IL-13 as predictive of olfactory function.
Nakayama et al., 2022 [58]	Interrogate endotype differences between White/non-Asian and Japanese CRSwNP populations.	Prospective multicenter cross-sectional study; whole-transcriptome and single-cell RNA sequencing of nasal polyps from 8 White and 9 Japanese CRSwNP patients, and 7 controls; independent qPCR validation set of 77 CRSwNP patients.	Unsupervised clustering analysis and Random Forest.	External qPCR validation and internal split-sample 500 iteration bootstrapping validation (63.2% training/36.8% testing).	Harabasz pseudo-F index, AUC, True positive rate, False positive rate, *p*-values.	Identified 2 major endotypes (type 2 and non-type 2) present in both cohorts but in differing ratios; scRNA-seq revealed prominent type 2 transcripts in specific epithelial cells and macrophages.
Romano et al., 2024 [59]	Determining the inflammatory profile and endotypic subgroups of CRSwNP patients across multiple centers.	Multicenter cross-sectional study; clinical data, tissue eosinophil/neutrophil counts, and 15 tissue cytokine concentrations from 166 CRSwNP patients and 80 controls across 15 Brazilian centers.	Hierarchical clustering and PCA.	Internal validation.	ROC curve analysis, AUC, *p*-values.	Brazilian CRSwNP patients exhibit a mixed inflammatory profile, with a ≥43 eosinophils/HPF cut-off effectively distinguishing high and low inflammation clusters with broader disease extent.
Sireci et al., 2024 [60]	Assess alignment of biologic therapy recommendations between human experts and AI.	Observational cohort study; clinical and laboratory (eosinophils, IgE) parameters of type 2 inflammation from 72 CRSwNP patients.	LLM application: ChatGPT.	Comparative expert-AI validation: ChatGPT (version 4.0, openAI, San Francisco, CA, USA) and multidisciplinary Rhinology Board.	Concordance percentage, Kappa coefficient, *p*-values.	ChatGPT demonstrated a 68% concordance with the Board (Kappa = 0.69); concordance was highest for dupilumab (79.6%).
Soler et al., 2021 [61]	Explore inflammatory endotypes in CRS using a phenotype-free approach.	Prospective multicenter case–control study; 26 biomarkers from olfactory cleft mucus in 198 subjects (128 CRS, 70 controls).	Agglomerative hierarchical clustering and PCA.	Internal validation.	Agglomeration coefficients, *p*-values.	Identified 10 clusters with distinct inflammatory signatures (e.g., type 2, mixed type 1/Th17); olfactory measures differed significantly across clusters.
Thorwarth et al., 2021 [62]	Predict eCRS using clinical and biomarker inputs.	Retrospective cohort study; peripheral eosinophil count, urinary leukotriene E4 level, and polyp status from 80 patients.	Binary logistic regression and ANN.	Internal validation (random 67:33 train–test split, and a surgeon-specific train–test split).	AUC, Sensitivity, Specificity, Accuracy, DeLong test.	ANN models achieved an AUC of 0.956 using surgeon-specific datasets, outperforming univariate predictors for eCRS diagnosis.
Tomassen et al., 2016 [63]	Identify inflammatory endotypes of CRS based solely on immune markers.	Multicenter case–control study; 14 tissue immune markers and clinical phenotypes from 226 CRS patients and 106 healthy controls across 10 European centers.	PCA, Ascendant hierarchical clustering, and Partitioning Around Medoids clustering.	Internal validation.	Mean silhouette width, Jaccard stability, Baker–Hubert Gamma, Hubert–Levin C index, Adjusted *p*-values.	Identified 10 clusters (4 IL-5-negative, 6 IL-5-positive) that correlated with and differentiated clinical phenotypes and asthma prevalence.
Turner et al., 2018 [64]	Define clinically relevant CRS endotypes using minimally invasive mucus analysis.	Prospective case–control study; 18 mucus-derived biological variables (cytokines), along with clinical/olfactory scores from 90 CRS patients and healthy controls.	Hierarchical cluster analysis and PCA.	Internal validation.	Within- and between-class variance, Variable communality, Eigenvalues, *p*-values.	Identified 6 clinically relevant endotype clusters based entirely on the minimally invasive mucus approach.
Viksne et al., 2024 [65]	Classify specific inflammatory endotypes in CRSwNP.	Cross-sectional study; 11 biomarkers (IL-1, IL-4, IL-6, etc.) in nasal polyp tissues from 35 individuals.	Hierarchical cluster analysis and PCA.	Internal validation.	Eigenvalues, Cumulative variance, *p*-values.	Identified 5 endotypes; neutrophilic and mixed neutrophilic/type 2 inflammation patterns were predominant.
Wang H. et al., 2024 [66]	Identify hub genes and associated pathways as diagnostic or therapeutic targets.	Retrospective database analysis (in silico) with in vivo validation; transcriptome data from two GEO datasets (CRSwNP patients and healthy controls), followed by qPCR validation on a murine CRSwNP model.	WGCNA and machine learning methods (LASSO, Random Forest, SVM-RFE).	Internal cross-validation (10-fold for LASSO, 5-fold for SVM-RFE), External validation (independent GEO dataset), and Experimental validation (in vivo mouse model qPCR).	AUC, Calibration plots, Decision curve analysis, Cross-validation error, Mean square error.	Identified 11 hub genes; validated BTBD10, ERAP1, GIPC1, and PEX6 as potential novel diagnostic or therapeutic targets.
Wang X. et al., 2023 [67]	Identify CRS endotypes based on inflammatory and remodeling factors.	Cross-sectional study; 16 inflammatory and remodeling factors from nasal mucosal/ polyp tissues of 128 CRS patients and 24 healthy controls.	Hierarchical clustering and Principal coordinate analysis.	Internal validation and Experimental validation.	Empirical FF, *p*-values.	Classified patients into 5 distinct clusters ranging from non-type 2 to high type 2; disease severity and recurrence increased from clusters 1 to 5.
Wang Z. et al., 2022 [68]	Predict type 2 CRSwNP using non-invasive nasal secretion biomarkers.	Cross-sectional study; clinical features, blood counts, and inflammatory biomarkers from nasal secretions and polyp tissues of 231 CRSwNP patients.	Partitioning Around Medoids clustering, Logistic regression, and Decision tree.	Internal validation.	AUC, Gini index, *p*-values.	The decision tree model (utilizing IL-5, blood eosinophils, and asthma status) demonstrated a high AUC of 0.90 for predicting type 2 CRSwNP.
Wu et al., 2021 [69]	Perform automated cellular phenotyping diagnosis of nasal polyps.	Retrospective and prospective cohort study; 24,625 whole-slide imaging patches from a development cohort of 179 patients, plus clinical data from 158 prospective and 116 retrospective CRSwNP patients.	Deep learning: (CNN using EfficientNet B5 architecture).	Internal validation (Random 3:1:1 split for training, validation, and independent testing sets).	MAE, RMSE, R-squared, EVS, *p*-values.	AICEP 2.0 accurately identified inflammatory cells (MAE for eosinophils: 1.64%); enabled classification of 4 clinically significant cellular phenotypes.
Zhou et al., 2025 [70]	Identify biomarkers related to macrophage polarization and metabolism.	Retrospective database analysis (in silico) with experimental validation; Transcriptome data from two GEO datasets focusing on macrophage polarization and metabolism-related genes	WGCNA, LASSO, and Random Forest.	Internal cross-validation (LASSO, Random Forest), External validation (independent GEO dataset), and Experimental validation (RT-qPCR).	AUC, Out-of-bag error, Binomial deviance, Calibration curve, *p*-values.	Identified ALOX5, HMOX1, and PLA2G7 as biomarkers (AUC > 0.7); a nomogram built on these exhibited superior diagnostic performance.

Abbreviations: AERD, Aspirin-exacerbated respiratory disease; AIC, Akaike information criterion; ANN, artificial neural network; AUC, area under the receiver operating characteristic curve; CDF, cumulative distribution function; ceRNA, competing endogenous RNA; CNN, convolutional neural network; CRS, chronic rhinosinusitis; CRSsNP, chronic rhinosinusitis without nasal polyps; CRSwNP, chronic rhinosinusitis with nasal polyps; ECP, eosinophil cationic protein; eCRS, eosinophilic chronic rhinosinusitis; ESS, endoscopic sinus surgery; EVS, explained variance score; GEO, Gene Expression Omnibus; GM-CSF, granulocyte-macrophage colony-stimulating factor; LASSO, least absolute shrinkage and selection operator; LLM, large language model; MAE, mean absolute error; mIoU, mean intersection over union; NERD, non-steroidal anti-inflammatory drug-exacerbated respiratory disease; NMDEs, nasal mucus-derived exosomes; PAF, platelet-activating factor; PCA, principal component analysis; QOL, quality of life; RMSE, root mean square error; ROC, receiver operating characteristic; scRNA-seq, single-cell RNA sequencing; SNOT-22, 22-item Sino-Nasal Outcome Test; SVM-RFE, support vector machine–recursive feature elimination; WGCNA, weighted gene co-expression network analysis.

### 3.3. Risk of Bias and Applicability Assessment

The model development and model evaluation of the 31 included studies were systematically appraised using the PROBAST+AI tool in Table 4.

#### 3.3.1. Model Development (Methodological Quality)

During the model development phase, the majority of studies demonstrated low concerns in Domain 1 (Participants and Data Sources), Domain 2 (Predictors), and Domain 3 (Outcomes). Clinical data sources, diagnostic criteria, and predictor measurements were generally clearly defined. Notably, Sireci et al. [60] evaluated the performance of a pre-trained ChatGPT model without model development or retraining; therefore, the PROBAST+AI development phase was considered not applicable for this study.

Quality concerns primarily emerged in Domain 4 (Analysis). Recognizing the distinct nature of unsupervised models, exploratory clustering and machine-learning studies were not penalized merely because they did not follow conventional supervised prediction-modelling pipelines. Studies using relatively adequate sample sizes together with clearly described and appropriate analytical strategies, such as Dorismond et al. [47], Liao et al. [53], and Wang Z. et al. [68], were therefore rated as having low concerns in the analysis domain.

In contrast, high concerns were assigned when explicit structural analytical limitations were identified. The most frequent concern involved a very small effective sample size combined with high-dimensional exploratory network or clustering analyses, or sequential data-driven feature selection, as observed in studies such as Divekar et al. [45], Ishino et al. [50], Wang H. et al. [66], Wang X. et al. [67], and Zhou et al. [70]. These features may lead to unstable analytical solutions and limited reproducibility. Other concerns included outcome-driven univariable predictor selection prior to model fitting, which introduces predictor selection bias and a high risk of overfitting (Guo et al. [48]), and highly restricted effective development units in deep-learning pipelines, such as Hsu et al. [49], in which model training was based on only 10 whole-slide images.

#### 3.3.2. Model Evaluation (Risk of Bias, ROB)

During the model evaluation phase, Domain 1 (Participants and Data Sources) was assessed primarily according to participant selection, exclusions, and attrition, rather than simply according to whether an independent external validation cohort was available. Most studies had evaluation populations that were appropriate for their prespecified target populations and were therefore rated as having low concerns in Domain 1. Chowdhury et al. [44] was the exception because only 62 of 147 enrolled participants had longitudinal postoperative follow-up, resulting in a substantially restricted evaluation subset and a potential selection/attrition concern.

Overall, 27 of 31 studies (87.1%) were rated as having high overall risk of bias in the model evaluation phase, primarily because of concerns in Domain 4 (Analysis and Performance Evaluation). Under our conservative operational rule, studies without independent external performance evaluation were generally assigned a high ROB in Domain 4 when model performance relied solely on apparent performance or internal validation procedures, including data splitting, cross-validation, or bootstrap resampling. Studies incorporating independent participant data for model evaluation, including Hsu et al. [49] and Nakayama et al. [58], generally demonstrated lower concerns regarding evaluation bias. In contrast, independent biomarker verification without applying and evaluating the complete prediction model in an independent dataset, as in Chen J. et al. [42], was not considered equivalent to external validation of a prediction model. Wang H. et al. [66] performed external validation using an independent GEO dataset; however, the limited validation sample size raised high concern in evaluation Domain 4, contributing to a high overall risk of bias judgement.

Notably, in accordance with the PROBAST+AI guidance, Lou et al. [55] was assigned a low overall ROB despite the absence of a strict external cohort. Although Domain 4 raised concerns under our conservative operational rule, the study used a relatively large dataset (N = 366), appropriate internal cross-validation, and showed no material concerns in the other evaluation domains. The overall ROB judgment therefore remained low, reflecting the consideration of the study’s evaluation methods as a whole rather than a mechanical aggregation of individual domain judgments.

#### 3.3.3. Applicability

Applicability concerns were generally low across the included studies for both the model development and evaluation phases. The participant populations, predictors, outcomes, and clinical tasks were considered sufficiently aligned with the intended scope of the present systematic review. No material applicability concerns were identified that would limit interpretation of the included studies within the scope of this review.

**Table 4:** Risk of bias and applicability assessment based on the PROBAST+AI guidelines.

Author, Year	Eval Type	Dev D1	Dev D2	Dev D3	Dev D4	Overall Quality	Dev APP (D1/2/3)	Overall Dev APP	Eval D1	Eval D2	Eval D3	Eval D4	Overall ROB	Eval APP (D1/2/3)	Overall Eval APP
Adnane et al., 2017 [40]	Apparent	Low	Low	Low	High	High	L/L/L	Low	Low	Low	Low	High	High	L/L/L	Low
Becker et al., 2025 [41]	Internal	Low	Low	Low	Low	Low	L/L/L	Low	Low	Low	Low	High	High	L/L/L	Low
Chen J. et al., 2022 [42]	External–Independent biomarker verification	Low	Low	Low	High	High	L/L/L	Low	Low	Low	Low	High	High	L/L/L	Low
Chen Z. et al., 2025 [43]	Apparent + external biomarker verification	Low	Low	Low	Low	Low	L/L/L	Low	Low	Low	Low	High	High	L/L/L	Low
Chowdhury et al., 2020 [44]	Apparent	Low	Low	Low	High	High	L/L/L	Low	High	Low	Low	High	High	L/L/L	Low
Divekar et al., 2015 [45]	Apparent	Low	Low	Low	High	High	L/L/L	Low	Low	Low	Low	High	High	L/L/L	Low
Divekar et al., 2017 [46]	Apparent	Low	Low	Low	High	High	L/L/L	Low	Low	Low	Low	High	High	L/L/L	Low
Dorismond et al., 2025 [47]	Apparent	Low	Low	Low	Low	Low	L/L/L	Low	Low	Low	Low	High	High	L/L/L	Low
Guo et al., 2021 [48]	Apparent	Low	Low	Low	High	High	L/L/L	Low	Low	Low	Low	High	High	L/L/L	Low
Hsu et al., 2024 [49]	External	Low	Low	Low	High	High	L/L/L	Low	Low	Low	Low	Low	Low	L/L/L	Low
Ishino et al., 2024 [50]	Apparent	Low	Low	Low	High	High	L/L/L	Low	Low	Low	Low	High	High	L/L/L	Low
Kim et al., 2021 [51]	Internal	Low	Low	Low	High	High	L/L/L	Low	Low	Low	Low	High	High	L/L/L	Low
Li et al., 2024 [52]	Internal	Low	Low	Low	High	High	L/L/L	Low	Low	Low	Low	High	High	L/L/L	Low
Liao et al., 2018 [53]	Apparent	Low	Low	Low	Low	Low	L/L/L	Low	Low	Low	Low	High	High	L/L/L	Low
Liu et al., 2022 [54]	Internal/Temporal	Low	Low	Low	High	High	L/L/L	Low	Low	Low	Low	High	High	L/L/L	Low
Lou et al., 2016 [55]	Internal	Low	Low	Low	Low	Low	L/L/L	Low	Low	Low	Low	High	Low	L/L/L	Low
Miyake et al., 2019 [56]	Apparent	Low	Low	Low	Low	Low	L/L/L	Low	Low	Low	Low	High	High	L/L/L	Low
Morse et al., 2019 [57]	Internal	Low	Low	Low	Low	Low	L/L/L	Low	Low	Low	Low	High	High	L/L/L	Low
Nakayama et al., 2022 [58]	External validation + internal resampling	Low	Low	Low	High	High	L/L/L	Low	Low	Low	Low	Low	Low	L/L/L	Low
Romano et al., 2024 [59]	Apparent	Low	Low	Low	Low	Low	L/L/L	Low	Low	Low	Low	High	High	L/L/L	Low
Sireci et al., 2024 [60]	External (pre-trained LLM)	N/A	N/A	N/A	N/A	N/A	N/A	N/A	Low	Low	Low	Low	Low	L/L/L	Low
Soler et al., 2021 [61]	Apparent	Low	Low	Low	Low	Low	L/L/L	Low	Low	Low	Low	High	High	L/L/L	Low
Thorwarth et al., 2021 [62]	Internal	Low	Low	Low	Low	Low	L/L/L	Low	Low	Low	Low	High	High	L/L/L	Low
Tomassen et al., 2016 [63]	Apparent	Low	Low	Low	Low	Low	L/L/L	Low	Low	Low	Low	High	High	L/L/L	Low
Turner et al., 2018 [64]	Apparent	Low	Low	Low	High	High	L/L/L	Low	Low	Low	Low	High	High	L/L/L	Low
Viksne et al., 2024 [65]	Apparent	Low	Low	Low	High	High	L/L/L	Low	Low	Low	Low	High	High	L/L/L	Low
Wang H. et al., 2024 [66]	External	Low	Low	Low	High	High	L/L/L	Low	Low	Low	Low	High	High	L/L/L	Low
Wang X. et al., 2023 [67]	Apparent	Low	Low	Low	High	High	L/L/L	Low	Low	Low	Low	High	High	L/L/L	Low
Wang Z. et al., 2022 [68]	Internal	Low	Low	Low	Low	Low	L/L/L	Low	Low	Low	Low	High	High	L/L/L	Low
Wu et al., 2021 [69]	Internal/Temporal	Low	Low	Low	Low	Low	L/L/L	Low	Low	Low	Low	High	High	L/L/L	Low
Zhou et al., 2025 [70]	External	Low	Low	Low	High	High	L/L/L	Low	Low	Low	Low	High	High	L/L/L	Low

Abbreviations: APP, application; Dev, development; Eval, evaluation; ROB, risk of bias. D1 domain: Participants and data sources. D2 domain: Predictors. D3 domain: Outcomes. D4 domain: Analyses.

### 3.4. AI and Machine Learning Applications

The AI applications in the 31 included studies were categorized into four main areas, according to their primary application. Fifteen studies (48.4%) focused on CRS classification and phenotyping. Nine studies (29.0%) involved prognostication and patient outcomes. Four studies (12.9%) were categorized under pharmacology and therapeutic optimization. Three studies (9.7%) focused on imaging and diagnostic analysis. As detailed in our PROBAST+AI evaluation (Section 3.3), 27 of the 31 included studies (87.1%) were assessed as having a high risk of bias in the model evaluation phase, primarily due to concerns in Domain 4 (Analysis and Performance Evaluation), particularly reliance on apparent performance or internal validation and limited independent evaluation of model performance. Consequently, throughout the following subsections, most reported performance metrics (e.g., high AUCs and prediction accuracies) reflect internally validated or apparent estimates. These headline figures should be interpreted with caution, as they may represent optimistic estimates of model performance.

#### 3.4.1. CRS Classification

While some deep learning algorithms applied to digital pathology also contribute to phenotypic classification (discussed separately in Section 3.4.2), the 15 studies detailed in this section mostly employed clustering algorithms on biomarker and clinical data to uncover inflammatory endotypes.

In 2016, a landmark study by Tomassen et al. identified 10 inflammatory endotype clusters of CRS through an unsupervised cluster analysis based on 14 cytokine and immune biomarkers in a phenotype-free approach. The results revealed a spectrum of immune responses, ranging from non-inflammatory profiles to high type 2 inflammation presentation, and were further matched with clinical phenotypes. They identified four clusters that exhibited low or undetectable levels of IL-5, ECP, IgE, and albumin, which are markers associated with type 2 inflammation, and corresponded with the CRSsNP phenotype and a low asthma prevalence. Among them, Cluster 1 showed no significant inflammatory elevation. Cluster 2 demonstrated a TNF-α/IL-22 profile. Cluster 3 was marked by IFN-γ and MPO/IL-8 elevation. Cluster 4 exhibited Th17-neutrophilic inflammation. The remaining six clusters showed high IL-5 and eosinophilic signatures, with further categorization based on IL-5 levels and comorbidity of asthma. Clusters 5 to 7 showed moderate IL-5 levels and mixed CRSsNP/CRSwNP features, while Clusters 8 to 10 displayed very high IL-5, IgE, and SE-IgE levels and were composed almost exclusively of CRSwNP patients with a high asthma prevalence. Clusters 9 and 10, particularly, had the highest IgE levels and universally expressed SE-IgE, indicating a strong superantigen-driven Th2 inflammation [63]. Romano et al. validated tissue cytokine profiles in a Brazilian multicenter cross-sectional study, where hierarchical clustering and principal component analysis revealed two main clusters of high and low inflammatory patterns. They determined that a tissue eosinophilia cut-off of 43 eosinophils per high-power field distinguished these two distinct inflammatory profiles [59].

Further studies expanded on tissue networks and remodeling factors. Given that a laboratory-based approach may not be practical in routine clinical settings, Divekar et al. [46] applied an unbiased network-mapping approach using a commercially available immunoassay panel for the endotyping of sinonasal tissue obtained from CRS patients and control subjects, which revealed three distinct cytokine–chemokine clusters in CRS patients. Cluster 1 was enriched for Th1/Th17-type markers. Cluster 2 was dominated by Th2-associated cytokines. Cluster 3 was characterized by elevated levels of chemokines and growth factors [46]. To study the association between endotypes and remodeling of CRS, Wang X. et al. identified 5 endotypes of CRS by integrating inflammatory and remodeling biomarkers, including cytokines, neutrophil factors, remodeling factors, MMP and fibronectin. Notably, Cluster 3 was characterized by low type 2 inflammation but exhibited high expression of neutrophil-associated factors, such as IL-8, G-CSF, MPO, and extracellular matrix proteins such as fibronectin, suggesting a neutrophil-dominant remodeling endotype. Cluster 5 demonstrated pronounced type 2 inflammation, accompanied by comparatively elevated levels of neutrophil-associated and tissue remodeling markers. Across Clusters 1 to 5, there was a progressive increase in the prevalence of nasal polyps, asthma, allergy, anosmia, aspirin sensitivity, and CRS recurrence [67]. Similarly, Viksne et al. utilized hierarchical cluster analysis to evaluate cytokines and antimicrobial peptides, classifying five specific inflammatory endotypes and observing that endotypes associated with neutrophilic inflammation or a mixed neutrophilic/type 2 pattern were predominant in their study population [65].

In addition to inflammatory pathways, machine learning has also been used in analyzing public transcriptomic databases and molecular subtypes. Chen Z. et al. applied machine learning algorithms to datasets from the Gene Expression Omnibus and classified CRSwNP patients into two distinct anoikis-related molecular subtypes characterized by different immune microenvironments. The models further identified four key anoikis-related genes, CDH3, PTHLH, PDCD4, and androgen receptor, with high diagnostic accuracy. Among these, androgen receptor and PTHLH were recognized as cluster-specific biomarkers [43]. Nakayama et al. investigated transcriptomic differences using whole-transcriptome and single-cell RNA sequencing (scRNA-seq). Their unbiased unsupervised clustering revealed that White and Japanese patients harbor the same major type 2 and non-type 2 endotypes, though with a statistically significant difference in the proportion of these endotypes between the two populations. There was a markedly higher prevalence of the type 2 endotype in White patients compared to Japanese patients. Furthermore, using droplet-based scRNA-seq, they mapped the cellular origins of newly identified signature transcripts, discovering that type 2-driving chemokines such as CCL13 and CCL18 were upregulated within CD163-high M2 macrophages, whereas cystatin SN (CST1) and CCL26 were expressed in basal, suprabasal, and secretory epithelial cells. Their random forest classifier further established that a select panel of these novel transcripts, particularly CCL13, CST1, and CCL18, could predict type 2 endotypes with an internally resampled AUC of 0.956; although the transcriptomic signatures were externally validated via an independent qPCR cohort, this specific model performance metric relies on internal bootstrapping [58]. Ishino et al. performed transcriptomic analysis using bulk RNA barcoding and sequencing. The hierarchical cluster analysis revealed that a cluster featuring severe type 2 inflammation is associated with upregulated platelet-activating factor (PAF) synthesis—the enzyme LPCAT1—and downregulated PAF degradation—the enzyme PAFAH2 [50].

Since the tissue-based assessment involves an invasive nature, Turner et al. defined CRS endotypes in a U.S. population entirely based on analysis of mucus collected via a minimally invasive approach. Similar to tissue-based results from a European population [63] but differing methodologically, they identified 6 inflammatory endotypes that differ substantially with respect to phenotype and disease behavior [64]. Similarly, Miyake et al. utilized nasal mucus-derived exosomes (NMDEs) to perform a liquid biopsy as an efficient alternative to traditional tissue sampling. Through cluster analysis, they demonstrate that a high Cystatin-2 expression level was associated with CRS comorbidities, and therefore, the cystatin level in NMDEs can predict CRS phenotype and disease severity, suggesting potential utility for treatment stratification [56]. Moreover, Becker et al. demonstrated the potential of machine learning for the discovery of non-invasive biomarkers in CRS phenotyping and endotyping. In a study on nasal mucus from 103 CRS patients, the authors applied complex algorithms, such as t-SNE, AdaBoost, and XGBoost, to a panel of 12 inflammatory proteins. Their models showed that mucus-based biomarkers had predictive performance for CRS phenotypes similar to conventional tissue-based methods. They discovered that IL-5 acted as the major driver for patient clustering, whereas periostin and cystatin-SA were important markers in epithelial- and tissue-derived signatures. Additionally, a four-biomarker panel of IL-5, IgE, IL-17, and periostin had the best predictive performance. These findings support the potential utility of AI-based analysis of non-invasive samples for CRS classification [41]. Wang Z. et al. established a pre-pruned decision tree model based on the levels of IL-5 in nasal secretion, blood eosinophil counts, and the asthma status of CRSwNP patients, yielding an internally validated AUC of 0.90 for predicting high levels of type 2 biomarkers [68].

Systemic and mixed clinical indicators were explored for CRS classification. Divekar et al. [47] applied unsupervised network analysis to assess serum biomarkers during acute exacerbations of CRSwNP, revealing a systemic increase in growth factors, specifically VEGF and GM-CSF [45]. Many studies have focused on identifying biomarkers for eosinophilic chronic rhinosinusitis (eCRS), but none have employed methods from the field of AI. Thorwarth et al. used supervised machine learning techniques, specifically logistic regression and artificial neural networks (ANN), to predict eCRS of patients who underwent endoscopic sinus surgery (ESS) for the treatment of CRS, based on 3 input variables, which are peripheral eosinophil count, urinary leukotriene E4 (uLTE4) level, and polyp status. The ANN models using the surgeon-specific dataset demonstrated predictive ability with a sensitivity of 92.3% (95% CI: 65.0–99.8) and a specificity of 85.7% (95% CI: 42.1–99.6) for the eCRS field [62].

Across clustering studies, the number of identified endotypes ranged from 2 to 10, with substantial variation in input features, clustering approaches, and criteria used to assess cluster stability or separation. Although several studies identified broadly overlapping inflammatory patterns, such as type 2, neutrophilic, and mixed inflammatory profiles, the proposed endotype structures were not directly comparable because they were derived from largely non-overlapping biological variables and heterogeneous analytical frameworks. Consequently, the available evidence demonstrates substantial molecular and inflammatory heterogeneity in CRS but does not yet establish a single reproducible AI-derived endotype classification framework across independent populations.

#### 3.4.2. Histopathological Image Analysis

To study the cellular phenotyping diagnosis of nasal polyps by whole-slide imaging (WSI), Wu et al. established an AI chronic rhinosinusitis evaluation platform 2.0 (AICEP 2.0) by applying a convolutional neural network (CNN)-based deep learning model. As the first AI for cellular phenotyping, AICEP 2.0 analyzed the proportion of inflammatory cells for each image and discovered four phenotypes of nasal polyps with different clinical characteristics. They also demonstrated that the percentage of eosinophils in peripheral blood was positively correlated with that in polyp tissue on WSI [69]. Similarly, Hsu et al. developed a CNN with query-driven multiple instance learning (MIL)-based automated eosinophil detector and achieved a sensitivity of 100.00% and a specificity of 90.48% [49].

For the purpose of examining the relationship between angiogenesis and the immune microenvironment, Liu et al. utilized a fully convolutional network (FCN) to quantify microvessels of tissues from CRS patients and revealed that angiogenesis was associated with type 2 inflammation. Additionally, they discovered a negative correlation between angiogenesis and local tissue expression of TNF-α and TGF-β [54].

#### 3.4.3. Pharmacology and Therapeutic Optimization

Four studies applied artificial intelligence and bioinformatics to identify novel molecular therapeutic targets and optimize pharmacologic treatments for CRSwNP.

Wang H. et al. applied multiple machine learning algorithms, including weighted gene co-expression network analysis (WGCNA), least absolute shrinkage and selection operator (LASSO) regression, random forest (RF), and SVM-RFE algorithms to identify hub genes associated with the pathogenesis of CRSwNP. They recognized gene *BTBD10*, *ERAP1*, *GIPC1*, and *PEX6* as key hub genes and potential novel therapeutic targets [66]. Similarly, Zhou et al. focused on macrophage polarization and metabolism and utilized WGCNA, LASSO, and RF algorithms to screen for candidate genes. They identified *ALOX5*, *HMOX1*, and *PLA2G7* as core biomarkers of CRSwNP. Additionally, using a drug–biomarker interaction network, they further predicted that selenium and the transcription factor FOXC1 are potential regulatory targets [70].

Exploring post-transcriptional regulation, Chen J. et al. constructed a competitive endogenous RNA (ceRNA) network using data mining and experimental verification. They identified a novel *MIAT*/*miR-125a*/*IRF4* regulatory axis, demonstrating that the long noncoding RNA *MIAT* acts as a sponge to regulate *IRF4* expression. This ceRNA axis was correlated with immune cell infiltration, specifically dendritic cells and M2 macrophages, offering further insight into novel molecular therapeutic targets for CRSwNP [42].

At the clinical decision-making level, Sireci et al. performed a novel application of large language models by using ChatGPT to guide the selection of biological therapies, including dupilumab, mepolizumab, and omalizumab, for patients with CRSwNP. The model showed a concordance percentage of 68% with the recommendations of the multidisciplinary Rhinology Board, indicating its potential to assist otolaryngologists in selecting biological therapy. However, they stated that it requires further training with large databases and integration with clinical data and image processing to enhance its diagnostic ability [60].

#### 3.4.4. Prognostication and Surgical Outcomes

Nine studies addressed prognostication and surgical outcomes, applying predictive models to forecast postoperative symptom improvement, long-term disease recurrence, and revision surgery risks.

Adnane et al. aimed to employ an unsupervised clustering method to identify which patient groups achieve the greatest improvement in QOL following ESS. They classified three distinct clusters of patients with CRS using unsupervised cluster analysis. All clusters showed significant clinical improvement, but there were significant differences in QOL outcomes between clusters, with factors like nasal polyps and mucosal eosinophilia endotype negatively impacting QOL improvement [40]. Similarly, Chowdhury et al. investigated the utility of baseline mucus cytokines in predicting postoperative 22-item Sino-Nasal Outcome Test (SNOT-22) scores using random forest and stepwise multivariate linear regression algorithms. They found that mucus IL-5 levels independently predicted postoperative SNOT-22 improvement, whereas IL-2 and TNF-α predicted postoperative worsening [44]. Focusing specifically on smell loss, Morse et al. applied machine learning algorithms, including hierarchical cluster analysis and random forest, to identify patterns of olfactory dysfunction in CRS patients, finding that anosmia is associated with a specific endotype characterized by severe nasal polyposis, tissue eosinophilia, and aspirin-exacerbated respiratory disease (AERD). Furthermore, IL-2, CT scores, and AERD were independently predictive of olfactory function. These findings indicated that a complex integration of inflammatory, clinical, and demographic factors contributes to olfactory loss in CRS patients [57].

Other models have been designed to predict disease trajectory and the need for revision surgery. Lou et al. identified five cellular phenotypes of CRSwNP and developed a clinical algorithm to predict recurrence, reporting an internally validated 98.5% polyp recurrence rate for a marked tissue eosinophilia cluster; although this model was assessed as having a low overall risk of bias due to its large sample size and robust internal procedures, the estimate still requires independent external confirmation for broader generalizability [55]. Similarly, Dorismond et al. utilized hierarchical clustering to analyze the cytokine profiles of 269 CRSwNP patients to predict time to recurrence. They identified six disease clusters and revealed a correlation between inflammatory endotypes and the time to recurrence. Two clusters, one exhibiting a mixed Type 1 and Type 3 inflammatory profile, including IFN-γ, IL-4, and IL-17A, and the other with elevated IL-12 and IL-21, showed the shortest time to recurrence. On the other hand, a cluster featuring innate and proinflammatory markers, including IL-1β, IL-6, and IL-8, showed the longest recurrence-free interval. These findings suggested that difficult-to-treat cases may require novel therapeutic strategies beyond Type 2 inflammation targets to prevent disease recurrence [47].

Identifying patients at high risk for difficult-to-treat or refractory CRS has also been a major focus of predictive modeling. Liao et al. integrated both clinical and molecular variables into a cluster analysis, identifying seven endotypes. They demonstrated that both severe eosinophilic and neutrophilic inflammation can lead to poor treatment outcomes, while a high IL-10 level cluster had no difficult-to-treat cases, suggesting the role of IL-10 in limiting inflammation [53]. To predict with a non-invasive method, Guo et al. constructed a prediction model using classification and regression tree (CART) and random forest algorithms. They found that the CCL17 levels of nasal secretion, hyposmia scores, allergic rhinitis comorbidity, and MIP-1β levels demonstrated an internally validated accuracy of 94% for predicting difficult-to-treat CRS [48]. For specific histologic subtypes, Kim et al. employed decision tree and random forest models to demonstrate that subepithelial neutrophil infiltration (human neutrophil elastase-positive cells), along with Lund–Mackay scores and endotype, acts as a cellular biomarker for predicting poor surgical outcomes of CRSwNP patients [51]. For non-eosinophilic CRSwNP, Li et al. utilized a random forest algorithm and stepwise logistic regression to evaluate clinical and biological variables. Their integrated model achieved an internally validated AUC of 0.89, identifying the biomarkers PDGF-β, PD-L1, MIP-3b, and PDGF-α as independent predictors of postoperative uncontrolled disease status in patients without significant eosinophilic infiltration [52].

## 4. Discussion

### 4.1. Summary of Principal Findings

This systematic review summarizes the current applications and potential of AI in advancing the understanding and clinical evaluation of CRS. Across the 31 included studies, AI-based approaches were used to characterize molecular and inflammatory endotypes and to analyze multidimensional data, including tissue transcriptomics, non-invasive biomarkers, digital pathology, and clinical variables. These applications identified heterogeneous inflammatory patterns, candidate therapeutic targets, and factors associated with postoperative outcomes, highlighting the potential of AI to support more detailed CRS phenotyping and risk stratification.

### 4.2. CRS Endotyping and Non-Invasive Diagnostics

Endotypic classification is based on underlying immunologic mechanisms, allowing a more precise identification of disease variants and their associated biomarkers [33]; also, understanding the cellular diversity in CRSwNP can help in diagnosis and the selection of therapeutic approaches, including the use of biologics [71]. The AI-driven clustering models reviewed in this study reveal a much more complex and heterogeneous spectrum [46,59,63,65,67]. These findings demonstrate the potential of biomarker-based clustering to identify immunologic profiles with potential clinical relevance and may provide a basis for future treatment stratification [33]. However, a critical examination of these models highlights a lack of consensus that must be confronted. The number of defined endotypes ranged from 2 to 10. This variation was accompanied by substantial differences in input features, clustering strategies, and criteria used to assess cluster separation or stability, limiting direct comparison and reproducibility across studies. The current literature presents a fragmented landscape of localized models rather than a universal CRS endotyping scheme. Therefore, while these models demonstrate the potential of AI in revealing immunologic profiles, their immediate clinical utility is hindered by this lack of standardization. Reconciling these divergent endotype schemes requires future large-scale multicenter studies to validate unified biomarker panels using standardized mathematical clustering metrics.

AI-based analyses of non-invasive specimens, including nasal secretions, mucus-derived exosomes, and serum, suggest the potential for the transition from invasive tissue biopsies, which are often limited by spatial heterogeneity across the sinonasal cavity [72,73], to non-invasive diagnostic strategies. Multiple studies applied complex algorithms, such as AdaBoost, XGBoost, and decision trees, to nasal secretions, mucus-derived exosomes, and serum to predict CRS endotypes [41,45,56,62,64,68]. By identifying biomarker panels, ML models have potential in disease endotyping in the outpatient setting, bypassing the need for invasive tissue sampling.

### 4.3. Accelerating Pharmacological Research and Therapeutic Optimization

AI can be applied in therapeutics for a dual purpose: accelerating drug discovery and optimizing clinical decision-making. The current treatment landscape for CRS is rapidly expanding toward targeted immunotherapies [26]. In this study, we reveal that machine learning frameworks have the ability to process high-dimensional transcriptomic data, searching for novel molecular targets and investigating complex regulatory networks [42,66,70]. From a translational perspective, these approaches may help prioritize candidate molecular targets and generate hypotheses for subsequent experimental validation and drug development [74,75].

Currently, using generative AI to guide the selection of monoclonal antibodies [60,76] shows potential to become an interactive bedside tool for clinicians. However, while large language models are promising for optimizing treatments, we must approach them with caution. Because these algorithms can still misinterpret data or generate “hallucinations,” their recommendations require strict verification by medical specialists before they can be safely used in real-world clinical practice [60].

### 4.4. Redefining Prognostication and Surgical Risk Assessment

FESS remains a solution for CRS refractory to medical therapy, but anticipating which patients will achieve disease control or suffer recurrent olfactory dysfunction, along with long-term disease trajectory, has relied on static clinical parameters [26,77]. By capturing complex, non-linear relationships among diverse variables, the reviewed approaches explored whether combinations of clinical, inflammatory, and molecular variables could improve risk stratification beyond conventional clinical indicators. [40,44,47,48,51,52,53,55,57]. A key pathophysiological insight synthesized from these models is the concern with the traditional Type 2-centric view of recurrence. While severe Type 2 inflammation is a well-established driver of surgical failure [55], AI models have identified mixed Type 1/3 or neutrophilic inflammatory profiles as associated with shorter time to recurrence or poorer postoperative outcomes [47,51]. This finding suggests that focusing exclusively on Type 2 pathways may be insufficient for a subset of patients.

In principle, validated prediction models could support individualized risk stratification and inform postoperative surveillance or treatment planning, changing the medical approach from simply reacting to problems to actively preventing them. However, the current evidence base does not yet establish that these models can reliably support such decisions in routine clinical practice [48,53,57].

**Figure 3:**
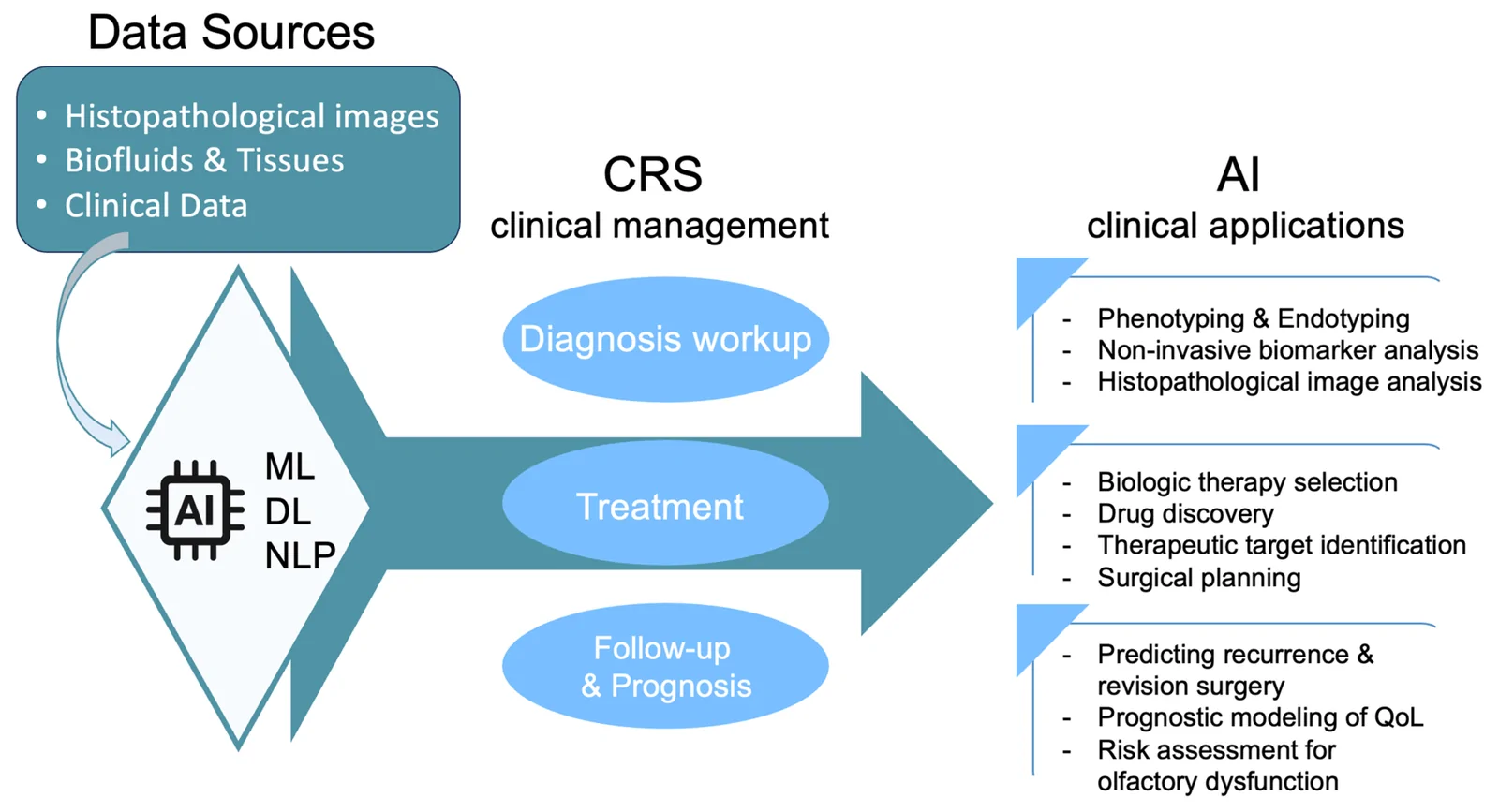
Integration of artificial intelligence (AI) into the clinical management of chronic rhinosinusitis (CRS). This figure illustrates the role of AI frameworks, including machine learning (ML), deep learning (DL), and natural language processing (NLP), in processing multi-modal data sources (histopathological images, biofluids, tissues, and clinical data) across the clinical management of CRS. AI has the potential to assist the diagnosis workup through clinical phenotyping, molecular endotyping, non-invasive biomarker analysis, and histopathological image analysis. In treatment, AI contributes to biologic therapy selection, drug discovery, therapeutic target identification, and surgical planning. During follow-up and prognosis, AI enables the prediction of disease recurrence and revision surgery, prognostic modeling of quality of life (QoL), and risk assessment for olfactory dysfunction.

Figure 3 demonstrates the potential role of AI in the clinical management of CRS for clinicians.

### 4.5. Challenges and Limitations of AI in CRS

Although AI has potential applications in medicine, some challenges and limitations remain. Current studies are often constrained by small sample size, single-center design, and various biases that limit the generalizability of the results. Data-related challenges, including limited quality of datasets, lack of standardization, and privacy or legal barriers to gathering the data, have further hindered model development and the reliability of generated results. At the technical level, many complex AI models remain difficult to interpret because their learned representations, parameters, and feature interactions are not readily understandable in human terms. Limited interpretability may complicate error analysis and assessment of model generalizability. Separately, overfitting remains a methodological concern in studies with small effective sample sizes and high-dimensional predictors, while generative AI systems may additionally produce hallucinated or unsupported outputs, still struggle with misinterpretation of information and data privacy issues, and need verification from specialists for those AI-generated findings [1,78,79].

Several limitations must be acknowledged, both at the review level and the study level. First, regarding our review methodology, the protocol for this systematic review was not prospectively registered in PROSPERO or a similar registry, which constitutes a methodological limitation. Furthermore, our search strategy was restricted to English-language, peer-reviewed articles across three major databases (PubMed, Web of Science, Embase), excluding grey literature, preprints, and trial registries. These restrictions introduce a potential language and publication bias. Because of the substantial heterogeneity in study designs, clinical tasks, and performance metrics across the included studies, a formal quantitative assessment of publication or reporting bias was not feasible; therefore, the risk of publication bias remains unquantified and must be acknowledged. Additionally, during data extraction, we primarily reported the metrics of the best-performing models from each study. Extracting only the best-performing models introduces selective reporting or selection bias at the review level and may lead to an overemphasis on the highest reported performance estimates, which might not be fully reproducible in prospective applications.

Second, at the study level, several included studies face potential limitations regarding model overfitting and cross-dataset confounding. For instance, some models were developed using sample sizes that were relatively small compared to the large number of analyzed features (e.g., evaluating a ~39-plex biomarker panel on fewer than 20 individuals [45]). In the absence of robust dimensionality reduction or independent external validation, these models may be susceptible to overfitting. The lack of reproducibility and comparability across the clustering schemes constitutes a major scientific limitation. Because studies utilize disparate input variables and validate their clusters using fundamentally non-comparable mathematical criteria, it remains unclear whether any of the proposed endotype schemes (which vary from 2 to 10 clusters) are reproducible across different populations. This central heterogeneity currently prevents the establishment of a consensus AI-driven CRS classification system. Additionally, for studies integrating independent microarray datasets (e.g., merging multiple GEO series), the management of batch effects was not consistently detailed. Unmitigated cross-dataset confounding could influence the reproducibility of the identified molecular targets.

Finally, the results of our PROBAST+AI assessment indicate a notable contrast between applicability and risk of bias in the current literature. As detailed in Table 4, the included studies consistently demonstrated low applicability concerns. This indicates that the populations, predictors, outcomes, and clinical tasks evaluated in the included studies were generally aligned with the prespecified scope of the review. However, 27 of the 31 studies (87.1%) were rated as having high overall risk of bias in the model evaluation phase, with concerns most frequently arising in Domain 4 (Analysis and Performance Evaluation). The common reliance on internal validation methodologies and the limited use of independent evaluation of model performance suggest that the reported performance metrics (e.g., high AUCs or accuracies) may be optimistic. Clinically, these concerns suggest that the reported performance estimates may be sensitive to the characteristics of the original development cohorts and may not generalize well to other populations or clinical settings. If these algorithms were deployed in different real-world healthcare settings, they could experience significant performance degradation. Therefore, while these AI models provide valuable translational insights into CRS, their generalizability remains uncertain, emphasizing the need for external validation before they can be integrated into routine clinical decision-making.

### 4.6. Future Directions

Current studies face several limitations, including dataset constraints, data privacy and ethical issues, and the variable accuracy of AI models. Future research should prioritize large, multicenter, and prospectively characterized cohorts with standardized clinical, molecular, and imaging data collection. For AI-derived endotypes, standardized input features and clustering methods, along with independent replication, are needed to assess reproducibility across populations. Predictive models require strict validation, independent external evaluation, calibration, and transparent reporting before clinical implementation. Prospective evaluation of multimodal AI systems in real-world settings is also needed to determine their clinical utility.

Looking forward, we envision the development of a well-established, comprehensive AI system for CRS clinical management that integrates personal profiles, clinical presentations, laboratory biomarkers, and macroscopic imaging data. This AI system could employ a nested architecture, with different AI algorithms being trained for different purposes within each dataset. These algorithms would then be integrated into a unified AI-assisted framework, with the potential to support rhinologists in daily practice by enhancing diagnostic accuracy, assisting in treatment decision-making, and optimizing patient outcomes through a data-driven, individualized approach.

## 5. Conclusions

This systematic review provides an overview of the current research findings, applications, and future potential of artificial intelligence (AI) in the clinical management of chronic rhinosinusitis (CRS). AI technologies, particularly machine learning (ML), deep learning (DL), and natural language processing (NLP), have been applied to CRS endotyping, histopathological image analysis, therapeutic target discovery, treatment selection, and outcome prediction.

AI-based clustering studies have revealed substantial inflammatory heterogeneity in CRS; however, the number of proposed endotypes, input features, and validation methods varied considerably across studies, limiting direct comparison and reproducibility. Future studies should prioritize standardized multicenter cohorts and independent replication to determine whether these endotype schemes can be reliably reproduced across populations. AI has also shown potential in digital pathology, molecular target discovery, and prognostic modeling, but the clinical utility of these applications remains to be established.

Importantly, 27 of 31 included studies (87.1%) were rated as having high overall risk of bias in the model evaluation phase, mainly because of limited independent evaluation of model performance. Therefore, reported performance estimates should be interpreted cautiously, and rigorous external validation in standardized, adequately sized, multicenter cohorts is needed before widespread clinical implementation.

## Figures and Tables

**Table 2:** Phenotypic and endotypic classifications of chronic rhinosinusitis (CRS) based on EPOS2020 [26].

Classification	Basis	Classification Groups		Key Features
Phenotype	Endoscopic presentation	CRSwNP		Bilateral nasal polyps, more common with Type 2 inflammation, asthma, AERD
		CRSsNP		No visible polyps, predominance of obstruction and pain symptoms
	Clinical course	AFRS		Polypoid CRS, eosinophilic mucin, non-invasive fungal hyphae, type I hypersensitivity to fungi
		AERD		Episodes of a respiratory reaction to aspirin or NSAIDs
		AECRS		Temporary worsening of CRS symptoms, may result from infection or exacerbation of asthma, return to baseline after treatment
		Difficult-to-treat rhinosinusitis		Failure to achieve control with appropriate medical therapy and sinus surgery
		Secondary CRS with systemic disease		CRS associated with cystic fibrosis, primary ciliary dyskinesia, granulomatosis with polyangiitis, or immunodeficiency
Endotype	Underlying immunologic mechanism	Non-Type 2	Type 1	IFN-γ, TNF-α, neutrophil-associated inflammation
			Type 3	IL-17, IL-22, neutrophil-dominant
		Type 2		IL-4, IL-5, IL-13, eosinophilic inflammation, IgE elevation

Abbreviations: AECRS, acute exacerbation of chronic rhinosinusitis; AERD, aspirin-exacerbated respiratory disease; AFRS, allergic fungal rhinosinusitis; IFN-γ, interferon-gamma; IgE, immunoglobulin E; IL, interleukin; NSAIDs, non-steroidal anti-inflammatory drugs; TNF-α, tumor necrosis factor-alpha.

## Data Availability

No new data were created or analyzed in this study. Data sharing is not applicable to this article.

## Supplementary Materials

The following supporting information can be downloaded at: https://www.mdpi.com/article/10.3390/ijms27198812/s1.

## Abbreviations

The following abbreviations are used in this manuscript:

AECRS	Acute Exacerbation of Chronic Rhinosinusitis
AERD	Aspirin-Exacerbated Respiratory Disease
AFRS	Allergic Fungal Rhinosinusitis
AI	Artificial Intelligence
ANN	Artificial Neural Network
AR	Acute Rhinitis
AUC	Area Under the Curve
AUROC	Area Under the Receiver Operating Characteristic curve
CART	Classification And Regression Tree
ceRNA	Competing Endogenous RNA
CNN	Convolutional Neural Network
CRS	Chronic Rhinosinusitis
CRSsNP	Chronic Rhinosinusitis without Nasal Polyps
CRSwNP	Chronic Rhinosinusitis with Nasal Polyps
DL	Deep Learning
eCRS	Eosinophilic Chronic Rhinosinusitis
EPOS	European Position Paper on Rhinosinusitis and Nasal Polyps
ESS	Endoscopic Sinus Surgery
FCN	Fully Convolutional Network
FESS	Functional Endoscopic Sinus Surgery
GEO	Gene Expression Omnibus
GM-CSF	Granulocyte-Macrophage Colony-Stimulating Factor
IFN-γ	Interferon-gamma
IgE	Immunoglobulin E
IL	Interleukin
LASSO	Least Absolute Shrinkage and Selection Operator
LLM	Large Language Model
MIL	Multiple Instance Learning
ML	Machine Learning
MPO	Myeloperoxidase
NLP	Natural Language Processing
NMDEs	Nasal mucus-derived exosomes
NSAIDs	Non-Steroidal Anti-Inflammatory Drugs
PAF	Platelet-Activating Factor
PCA	Principal Component Analysis
PRISMA	Preferred Reporting Items for Systematic Reviews and Meta-Analyses
PROBAST+AI	Prediction model Risk Of Bias Assessment Tool for AI
QOL	Quality Of Life
RF	Random Forest
RL	Reinforcement Learning
ROC	Receiver Operating Characteristic
scRNA-seq	Single-cell RNA sequencing
SNOT-22	22-item Sino-Nasal Outcome Test
SVM-RFE	Support Vector Machine-Recursive Feature Elimination
TNF-α	Tumor Necrosis Factor-alpha
t-SNE	t-distributed Stochastic Neighbor Embedding
VEGF	Vascular Endothelial Growth Factor
WGCNA	Weighted Gene Co-expression Network Analysis
WSI	Whole Slide Image
